# Development and validation of a nomogram for predicting hemoptysis recurrence in cystic bronchiectasis patients following bronchial artery embolization

**DOI:** 10.3389/fmed.2025.1582008

**Published:** 2025-05-15

**Authors:** Yuxin Duan, Weifan Sui, Zefeng Cai, Yimao Xia, Jianyun Li, Jianhua Fu

**Affiliations:** Department of Interventional Radiology, The Affiliated People's Hospital of Jiangsu University, Zhenjiang, China

**Keywords:** cystic bronchiectasis, nomogram, hemoptysis, bronchial artery embolization, recurrence prediction

## Abstract

**Background:**

Hemoptysis is a life-threatening manifestation frequently observed in patients with cystic bronchiectasis (CB), a radiologically defined subtype of bronchiectasis. Bronchial artery embolization (BAE) is widely employed as an effective interventional therapy for controlling hemoptysis. Despite its clinical utility, the risk of recurrence remains high, particularly in patients with CB. Currently, no reliable predictive model specifically targeting CB-related hemoptysis recurrence following BAE has been established, highlighting the need for a tailored prognostic tool in this population.

**Objective:**

This study aimed to develop and validate a model to predict the recurrence of hemoptysis in CB patients following BAE, enabling individualized clinical management and prevention strategies.

**Methods:**

A retrospective study was conducted on 111 CB patients who underwent BAE between January 2015 and June 2020. Clinical, radiological, and laboratory data were collected for analysis. Least absolute shrinkage and selection operator (LASSO) regression was applied to identify relevant predictive variables, followed by multivariable Cox proportional hazards regression to determine independent prognostic factors. Based on these predictors, a nomogram was constructed. Its performance was assessed using the concordance index (C-index), receiver operating characteristic (ROC) curves, area under the curve (AUC), calibration plots, and decision curve analysis (DCA).

**Results:**

Five independent predictors were identified: history of hemoptysis (HR = 3.42, 95% CI: 1.64–7.12, *p* = 0.001), diabetes (HR = 15.0, 95% CI: 4.69–48.1, *p* < 0.001), pleural thickening (HR = 3.78, 95% CI: 1.07–13.4, *p* = 0.039), prolonged hospitalization (HR = 1.99, 95% CI: 1.08–3.67, *p* = 0.028), and positive sputum culture (HR = 2.29, 95% CI: 1.26–4.19, *p* = 0.007). The nomogram showed good discriminatory ability, with AUCs of 0.778, 0.797, and 0.829 at 1-, 2-, and 3-year follow-ups, respectively. The integrated Brier score was 0.147, reflecting good overall accuracy. Time-dependent AUC and C-index curves further confirmed the model’s prognostic robustness. Calibration plots demonstrated close agreement between predicted and observed recurrence, and decision curve analysis indicated favorable clinical utility. Recurrence-free time was significantly shorter in the high-risk group (*p* < 0.0001).

**Conclusion:**

The nomogram is a reliable tool for predicting hemoptysis recurrence in CB patients after BAE. It facilitates early identification of high-risk patients, enabling timely, targeted interventions and improved outcomes.

## Introduction

Hemoptysis is a critical clinical manifestation of various respiratory disorders, posing significant risks such as asphyxia and acute blood loss. Bronchiectasis is one of the most frequent underlying etiologies of hemoptysis. In the progression of bronchiectasis, repeated airway infections lead to structural airway damage, leading to hemoptysis in 23–52% of patients that require prompt clinical management (1–5). BAE is an established and effective therapeutic option (6–8). Although BAE is a therapeutic option for managing hemoptysis, its recurrence rate in bronchiectasis patients remain high, ranging from 10 to 57% (3).

Previous studies have analyzed and modeled the risk factors for recurrence following BAE treatment in bronchiectasis patients (2, 9, 10). However, the prognosis of hemoptysis varies among different types of bronchiectasis, as classified by the Reid classification (11). Patients with CB are more likely to experience hemoptysis relapse and have poorer prognoses compared to those with cylindrical or tubular bronchiectasis (9, 12). Nevertheless, limited studies have specifically addressed the prediction of post-BAE recurrence in this population, underscoring the need for a dedicated prognostic model. In this study, we consolidated a relatively large dataset of CB patients who underwent BAE and developed a model to predict recurrence risk after BAE. This model facilitates individualized prevention and timely clinical intervention, potentially improving patient prognosis.

## Materials and methods

This retrospective study was approved by our institutional ethics review board, the requirement for patients’ informed consent was waived by the same ethics committee that approved the study. The indications and procedures for BAE followed the guidelines of the Society of Interventional Radiology (13). All procedures performed in studies involving human participants were in accordance with the ethical standards of the Affiliated People’s Hospital of Jiangsu University and/or national research committee and with the 1964 Helsinki declaration and its later amendments or comparable ethical standards.

### Patient criteria

This retrospective study analyzed patients with CB-related hemoptysis who underwent BAE at the Affiliated People’s Hospital of Jiangsu University between January 2015 and June 2020. Diagnoses of CB were confirmed using high-resolution computed tomography (HRCT) based on the Reid classification, which categorizes bronchiectasis into cystic and non-cystic forms, the latter subdivided into cylindrical and varicose types (14). Inclusion criteria were: (1) age ≥18 years, and (2) hemoptysis associated with idiopathic CB per Reid classification. Exclusion criteria were: (1) hemoptysis not related to CB; (2) technical or clinical failure of BAE; (3) previous history of BAE or pulmonary resection; and (4) incomplete data or loss to follow-up.

**Figure 1:**
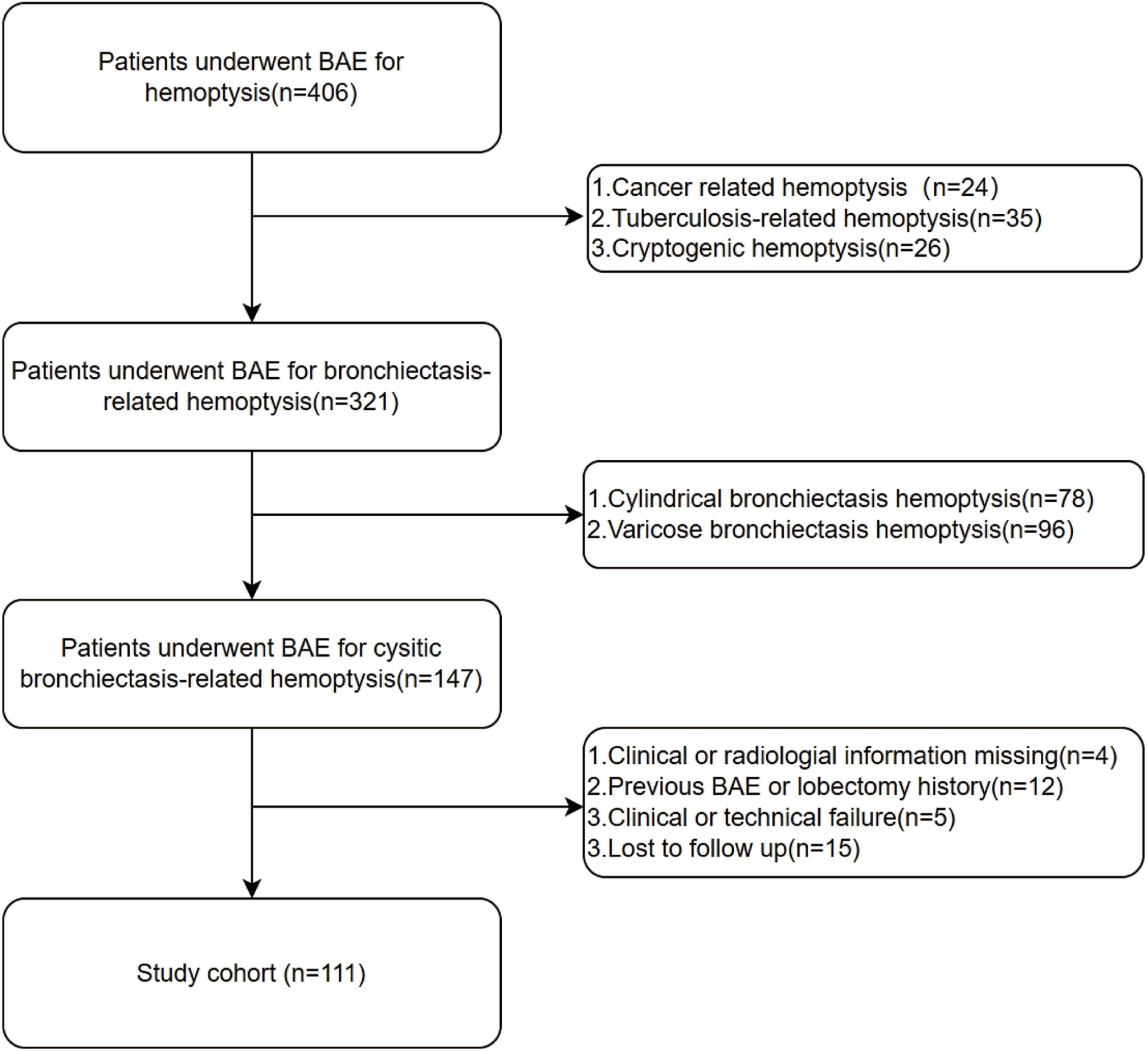
Flowchart for the selection procedure for cystic bronchiectasis patients with hemoptysis treated by bronchial artery embolization.

A total of 111 patients who met the inclusion criteria were included in the study. The flowchart of patient enrollment is shown in Figure 1. The collected data included clinical characteristics, peripheral blood samples, and radiological features. The detailed demographic characteristics are presented in Table 1. Hemoptysis severity is classified based on the amount of blood expectorated: mild (≤100 mL/day), moderate (100–300 mL/day), or severe (massive, >300 mL/day) (3). Clinical success is defined as the complete cessation of hemoptysis or its reduction to minimal levels (<10 mL/day) after BAE. Clinical failure is defined as recurrence of hemoptysis or death due to hemoptysis within 24 h post-BAE. Technical success refers to successful embolization of abnormal vessels during the BAE procedure, while technical failure refers to incomplete embolization or failure to identify abnormal arteries. Hemoptysis recurrence is defined as a total daily bleeding volume >30 mL/day after BAE (15). Peripheral blood samples were collected from the patients before BAE procedure. Positive sputum culture was defined as the detection of any pathogenic microorganism in sputum samples obtained prior to BAE, following standard microbiological culture procedures. Hemoptysis history was defined as any documented history of hemoptysis occurring within 6 months prior to BAE procedure, based on patient self-report and medical records. Diabetes was defined according to the American Diabetes Association (ADA 2020) criteria: fasting plasma glucose ≥126 mg/dL (7.0 mmol/L), HbA1c ≥ 6.5%, or a clinical diagnosis requiring antihyperglycemic treatment (16). Pleural thickening greater than 3 mm was considered pathological (17). Irreversible parenchymal destruction, characterized by diffuse adhesions and large cavities, was classified as lung destruction (18).

**Table 1:** Baseline characteristics of the study patients (*N* = 111).

Recurrence	NO	YES
	*N =* 57	*N =* 54
Gender		
Female	16 (28.1%)	27 (50.0%)
Male	41 (71.9%)	27 (50.0%)
Age (years)		
< 65	30 (52.6%)	30 (55.6%)
> =65	27 (47.4%)	24 (44.4%)
Hemoptysis volume (ml/d)		
Massive	1 (1.75%)	3 (5.56%)
Mild	32 (56.1%)	29 (53.7%)
Moderate	24 (42.1%)	22 (40.7%)
Hemoptysis history		
NO	41 (71.9%)	20 (37.0%)
YES	16 (28.1%)	34 (63.0%)
Smoking history		
NO	34 (59.6%)	43 (79.6%)
YES	23 (40.4%)	11 (20.4%)
SI		
< 200	35 (61.4%)	43 (79.6%)
> =200	22 (38.6%)	11 (20.4%)
Hypertension		
NO	40 (70.2%)	43 (79.6%)
YES	17 (29.8%)	11 (20.4%)
CHD		
NO	53 (93.0%)	50 (92.6%)
YES	4 (7.02%)	4 (7.41%)
Diabetes		
NO	55 (96.5%)	49 (90.7%)
YES	2 (3.51%)	5 (9.26%)
LOB		
< 3	35 (61.4%)	16 (29.6%)
> =3	22 (38.6%)	38 (70.4%)
CT bronchiectasis score		
< 10	46 (80.7%)	23 (42.6%)
> =10	11 (19.3%)	31 (57.4%)
NOC		
< 3	37 (64.9%)	27 (50.0%)
> =3	20 (35.1%)	27 (50.0%)
LOC		
< 3.3	39 (68.4%)	22 (40.7%)
> =3.3	18 (31.6%)	32 (59.3%)
SPSs		
Absent	40 (70.2%)	31 (57.4%)
Present	17 (29.8%)	23 (42.6%)
Sputum culture		
Negative	50 (87.7%)	30 (55.6%)
Positive	7 (12.3%)	24 (44.4%)
NBSA		
Absent	50 (87.7%)	36 (66.7%)
Present	7 (12.3%)	18 (33.3%)
Pleural thickening		
Absent	9 (15.8%)	3 (5.56%)
Present	48 (84.2%)	51 (94.4%)
CRP		
<3.47	38 (66.7%)	21 (38.9%)
> = 3.47	19 (33.3%)	33 (61.1%)
WBC (10^9^/L)		
<7.7	32 (56.1%)	27 (50.0%)
> = 7.7	25 (43.9%)	27 (50.0%)
NEUP		
<0.77	37 (64.9%)	26 (48.1%)
> = 0.77	20 (35.1%)	28 (51.9%)
RBC (10^12^/L)		
<4.0	22 (38.6%)	27 (50.0%)
> = 4.0	35 (61.4%)	27 (50.0%)
HGB (g/L)		
<116	20 (35.1%)	28 (51.9%)
> = 116	37 (64.9%)	26 (48.1%)
PLT (10^9^/L)		
<162	32 (56.1%)	19 (35.2%)
> = 162	25 (43.9%)	35 (64.8%)
Albumin (g/L)		
<37.9	25 (43.9%)	29 (53.7%)
> = 37.9	32 (56.1%)	25 (46.3%)
TP (g/L)		
<66.1	36 (63.2%)	26 (48.1%)
> = 66.1	21 (36.8%)	28 (51.9%)
PT(s)		
<11.2	24 (42.1%)	18 (33.3%)
> = 11.2	33 (57.9%)	36 (66.7%)
APTT(s)		
<26.9	23 (40.4%)	22 (40.7%)
> = 26.9	34 (59.6%)	32 (59.3%)
Duration of hospitalization (days)		
<11	42 (73.7%)	25 (46.3%)
> = 11	15 (26.3%)	29 (53.7%)
Vasoconstrictor use		
NO	12 (21.1%)	13 (24.1%)
YES	45 (78.9%)	41 (75.9%)

NBSAs, Non bronchial systemic arteries; SI, Smoking index; SPSs, Systemic arterial-pulmonary circulation shunts; CHD, Chronic heart disease; LOB, Lobes of bronchiectasis; NOC, Number of culprit bronchial arteries; LOC, Length of culprit bronchial arteries; CRP, C-Reactive protein; NEUP, Neutrophil Percentage; RBC, Red Blood Cell; HGB, Hemoglobin; PLT, Platelet; TP, Total protein; PT, Prothrombin time; APTT, Activated partial thromboplastin time.

### Management and follow-up

All patients underwent chest HRCT and CT angiography (CTA) prior to BAE to identify pulmonary infection and locate potential culprit vessels, enabling more precise targeting and embolization during the procedure. BAE indications and procedures adhered to the guidelines of the Society of Interventional Radiology (13). The BAE procedure for each patient was performed under local anesthesia via a puncture in the right femoral artery. Abnormal vessels in the aortic arch were initially visualized by angiography with a 5F pigtail catheter. Angiography of the culprit arteries, including bronchial and non-bronchial systemic arteries (NBSAs), was performed using a 5F Cobra 3 or 5F left gastric catheter. The angiographic features of culprit vessels included vascular dilatation, tortuosity, increased vascularity, formation of bronchial artery-pulmonary vascular fistulas, and contrast extravasation. Embolization materials were selected using a 2.7F or 2.4F microcatheter (2.7F; Terumo Medical Corp., Japan; or 2.4F; Boston Scientific, USA), with injection at the selected vessel diameter using 150–350 μm or 350–560 μm polyvinyl alcohol (PVA) particles (Hangzhou Alicon Pharmaceutical Co., Ltd., Zhejiang, China). Only two patients with large culprit vessels required Microcoils (Cook, USA) embolization. The technical endpoint was defined as the complete embolization of all identified culprit vessels. Follow-up was performed through telephone or outpatient visits, with radiological re-examination as necessary. Recurrence time was measured from the date of the BAE procedure until the recurrence of hemoptysis.

### Model construction and assessment

During feature selection, we incorporated clinical expertise and the literature on the etiology and pathology of bronchiectasis-related hemoptysis. Based on these insights and predefined inclusion and exclusion criteria, we ultimately included 31 variables in the study. To facilitate the construction and visualization of the nomogram, cutoff values for continuous variables were derived from ROC analysis based on the maximum Youden Index (19), as shown in Supplementary Table S1.

The study employed LASSO regression to identify significant clinical predictors while excluding irrelevant factors (20). Univariable Cox regression analysis was first performed on the 12 variables selected by LASSO. Variables with *p* < 0.1 were then entered into a multivariable Cox regression analysis. Based on the results of the multivariable analysis, a nomogram was constructed to predict hemoptysis recurrence at 1-, 2-, and 3-year intervals. The performance of the nomogram was compared against models derived from its individual constituent variables. The integrated Brier score, time-dependent ROC curves and time-dependent C-index curves were used to evaluate the nomogram’s goodness of fit and discriminative performance. Calibration curves were plotted to assess predictive accuracy, while DCA was conducted to determine clinical utility. Based on the nomogram-derived risk stratification, recurrence probabilities were estimated using the Kaplan–Meier method and compared between groups using log-rank tests.

### Statistical analysis

ALL Categorical variables were described as frequencies (percentages). Data analysis, model development, and evaluation were all performed using R software (Version 4.3.2) for Windows. Lasso regression was conducted using the *glmnet* package. Univariable and multivariable Cox regression analyses were performed using the *survival* package. The nomogram and calibration curve were plotted using the *rms* package. Time-dependent ROC, time-dependent AUC, and time-dependent C-index curves were plotted using the *timeROC* and *ggplot2* packages, and AUC was also calculated. The integrated Brier score was calculated using the inverse probability of censoring weighting (IPCW) method implemented in the *riskRegression* package. The DCA curve was plotted using the *ggDCA* package. Kaplan–Meier survival analysis and cut-off value calculation were performed using the *survminer* and *survival* packages. A *p*-value < 0.05 was considered statistically significant.

## Results

### Characteristics of patients

A total of 111 patients with CB who underwent BAE at the First People’s Hospital of Zhenjiang between January 2015 and June 2020 were enrolled (Table 1). The cohort included 68 males (61.3%) and 43 females (38.7%). Diabetes mellitus and hypertension were present in 7 (6.3%) and 28 (25.2%) patients, respectively. A history of smoking was documented in 34 patients (30.5%), and 50 patients (45.0%) had experienced hemoptysis prior to BAE. NBSAs were identified in 25 patients (22.5%), and SPSs in 40 patients (36.0%). Among the 31 patients (27.9%) with positive sputum cultures, *Pseudomonas aeruginosa* was identified in 20 cases, *Candida albicans* in 2 cases, and *Klebsiella pneumoniae* in 9 cases.

### Features selected in models

**Figure 2:**
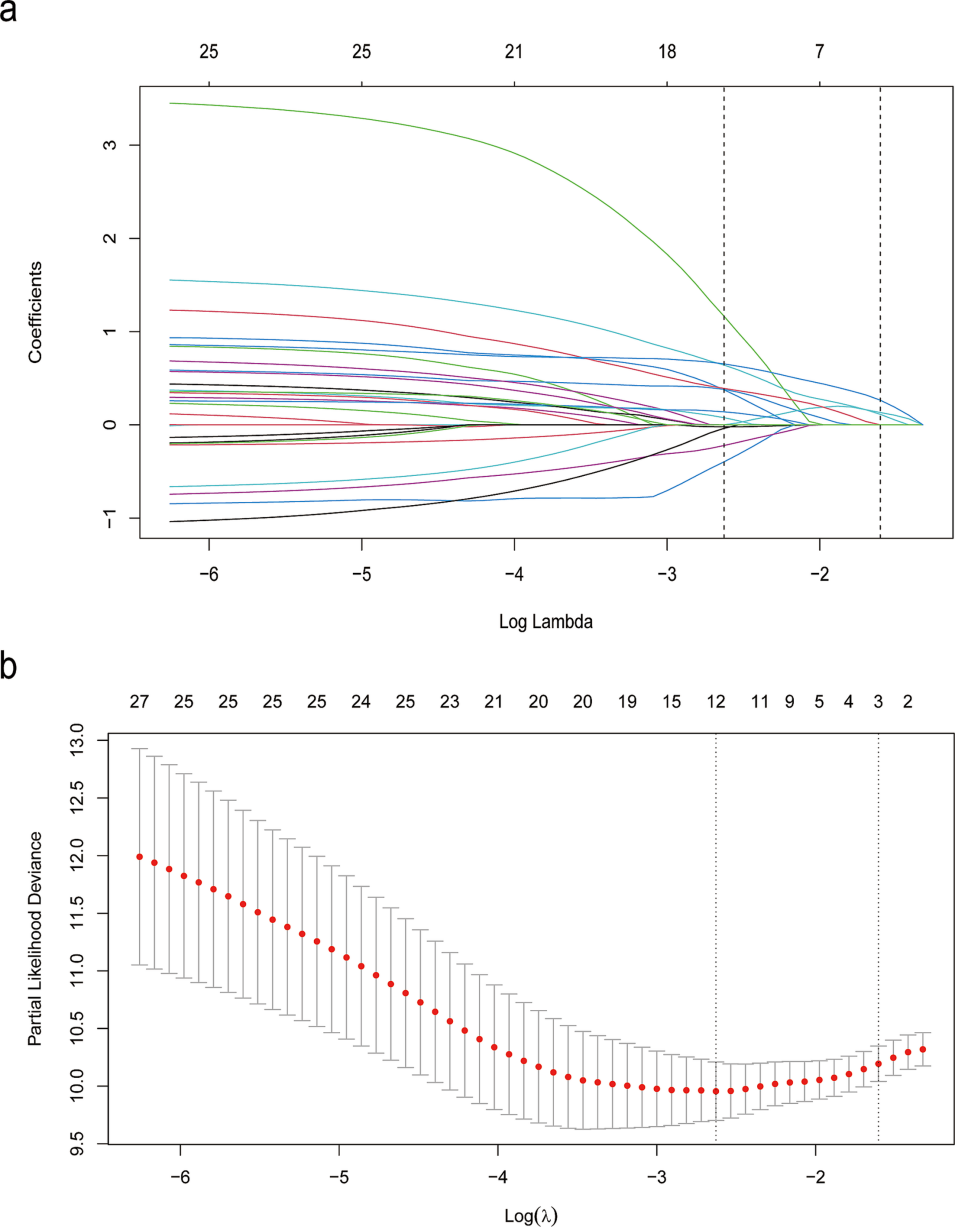
LASSO binary logistic regression results. **(a)** Coefficient paths across varying levels of the regularization parameter λ, illustrating the selection of demographic and clinical features. **(b)** Cross-validation plot showing the relationship between the λ parameter and model performance.

LASSO regression was employed to automatically select predictive features (Figure 2). This method minimizes binomial deviance in the loss function by tuning the regularization parameter lambda (*λ*), thereby shrinking some coefficients to zero and excluding non-contributing variables. Of the 31 candidate features, 12 were retained at the optimal shrinkage parameter (lambda. Min = 0.05998), including sex, history of hemoptysis, smoking history, hemoptysis volume, diabetes, NOB, NOC, pleural thickening, PLT, CRP, duration of hospitalization, and sputum culture results. These 12 variables were subsequently entered into a multivariable Cox proportional hazards model. Five variables emerged as statistically significant predictors of hemoptysis recurrence: history of hemoptysis (HR = 3.42, 95% CI: 1.64–7.12, *p* = 0.001), diabetes (HR = 15.00, 95% CI: 4.69–48.10, *p* < 0.001), pleural thickening (HR = 3.78, 95% CI: 1.07–13.40, *p* = 0.039), hospitalization duration ≥11 days (HR = 1.99, 95% CI: 1.08–3.67, *p* = 0.028), and positive sputum culture (HR = 2.29, 95% CI: 1.26–4.19, *p* = 0.007), as shown in Table 2. To assess potential multicollinearity, we conducted a variance inflation factor (VIF) analysis for the variables included in the final model. All VIF values were below 5, with a maximum of 1.18 (Supplementary Figure S1), indicating negligible multicollinearity among predictors (21).

**Table 2:** Univariate and multivariate analyses of the variables selected by Lasso regression.

	Univariate			Multivariate		
Characteristic	HR	95% CI	*p*-value	HR	95% CI	*p*-value
Gender						
Female	—	—		—	—	
Male	0.54	0.32, 0.92	**0.025**	0.90	0.47, 1.72	0.752
Hemoptysis history						
NO	—	—		—	—	
YES	2.93	1.68, 5.14	**<0.001**	3.42	1.64, 7.12	**0.001**
Smoking history						
NO	—	—		—	—	
YES	0.50	0.26, 0.97	**0.040**	0.50	0.22, 1.15	0.102
Pleural thickening	2.72	0.85, 8.76	0.093	3.78	1.07, 13.4	**0.039**
Hemoptysis volume(ml/d)						
Massive	—	—		—	—	
Mild	0.34	0.10, 1.11	0.074	1.81	0.50, 6.54	0.368
Moderate	0.30	0.09, 1.02	0.053	0.45	0.12, 1.60	0.216
Hypertension						
NO	—	—				
YES	0.76	0.39, 1.47	0.418			
CRP						
< 3.47	—	—		—	—	
> =3.47	2.04	1.18, 3.52	**0.011**	1.54	0.82, 2.91	0.183
Diabetes						
NO	—	—		—	—	
YES	2.73	1.08, 6.88	**0.034**	15.0	4.69, 48.1	**<0.001**
PLT(10^9^/L)						
< 162	—	—		—	—	
> =162	1.91	1.09, 3.34	**0.024**	1.46	0.80, 2.66	0.215
LOB						
< 3	—	—		—	—	
> =3	2.70	1.50, 4.88	**<0.001**	2.05	0.94, 4.47	0.070
Duration of hospitalization(days)						
< 11	—	—		—	—	
> =11	2.40	1.40, 4.11	**0.001**	1.99	1.08, 3.67	**0.028**
Sputum culture						
Negative	—	—		—	—	
Positive	3.35	1.93, 5.81	**<0.001**	2.29	1.26, 4.19	**0.007**

HR, Hazard Ratio, CI, Confidence Interval. Bold values indicate statistical significance (*p* < 0.1 in univariate analysis; *p* < 0.05 in multivariate analysis).

### Construction and validation of the nomogram

**Figure 3:**
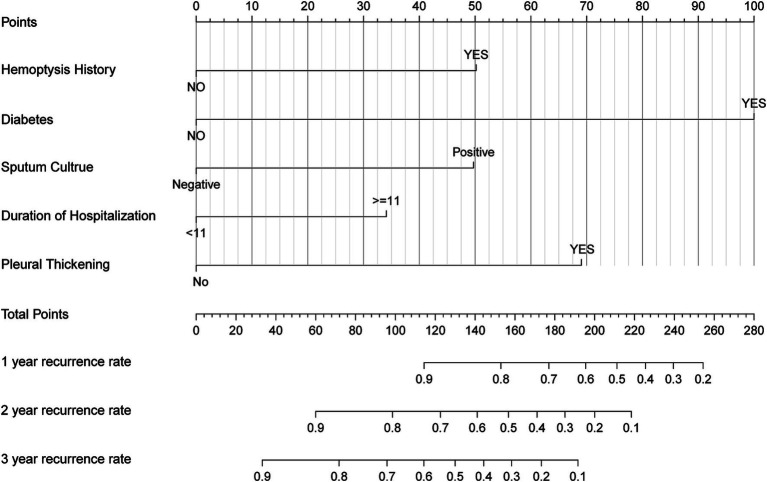
Nomogram based on history of hemoptysis, diabetes, pleural thickening, duration of hospitalization, sputum culture. The nomogram was used by summing the points identified on the points scale for each prognostic factor. The total points projected on the bottom scales match the probability of 1-, 2-, and 3-year recurrence of patients.

A nomogram was developed to predict the probability of hemoptysis recurrence at 1, 2, and 3 years in CB patients treated with BAE, based on five independent prognostic factors. Each variable was assigned a point value by projecting vertically from the specific factor value to the “Points” axis of the nomogram (Figure 3). The total score, obtained by summing the individual points, corresponds to predicted recurrence probabilities at 1, 2, and 3 years, as indicated by a vertical projection to the corresponding scales. A higher total point value reflects a greater risk of recurrence.

**Figure 4:**
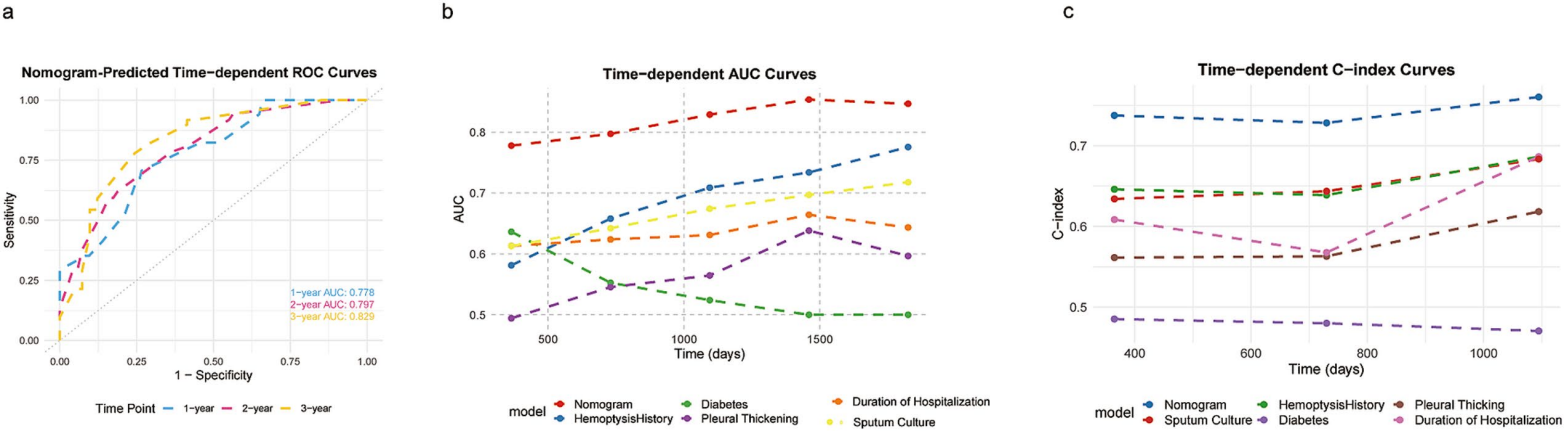
ROC curves of nomogram model to predict 1-, 2-, and 3-year recurrence rates of CB patients post BAE **(a)**. Time-dependent C-index **(b)** and time-dependent AUC curves **(c)** of nomogram compared with its own five variables.

The nomogram was validated by assessing its discrimination and calibration through 1,000 bootstrap resampling. Discrimination ability was evaluated using the C-index, ROC curves, AUC values, and integrated Brier score. Time-dependent ROC curves, AUC curves, and C-index curves were plotted for the nomogram and five alternative models (Figure 4). The AUC values for predicting recurrence at 1-year, 2-year, and 3-year intervals were 0.778 (95% CI: 0.667–0.873), 0.797 (95% CI: 0.67–0.886), and 0.829 (95% CI: 0.661–0.908), respectively; the resampled AUC values were 0.781 (95% CI: 0.77–0.792), 0.803 (95% CI: 0.795–0.812), and 0.831 (95% CI: 0.823–0.839), respectively, as shown in Supplementary Figure S2. Additionally, the integrated Brier score of 0.147 further supports the nomogram’s robust predictive performance, indicating low overall prediction error. In summary, these results underscore the nomogram’s superior ability to predict post-BAE recurrence outcomes in CB patients.

**Figure 5:**
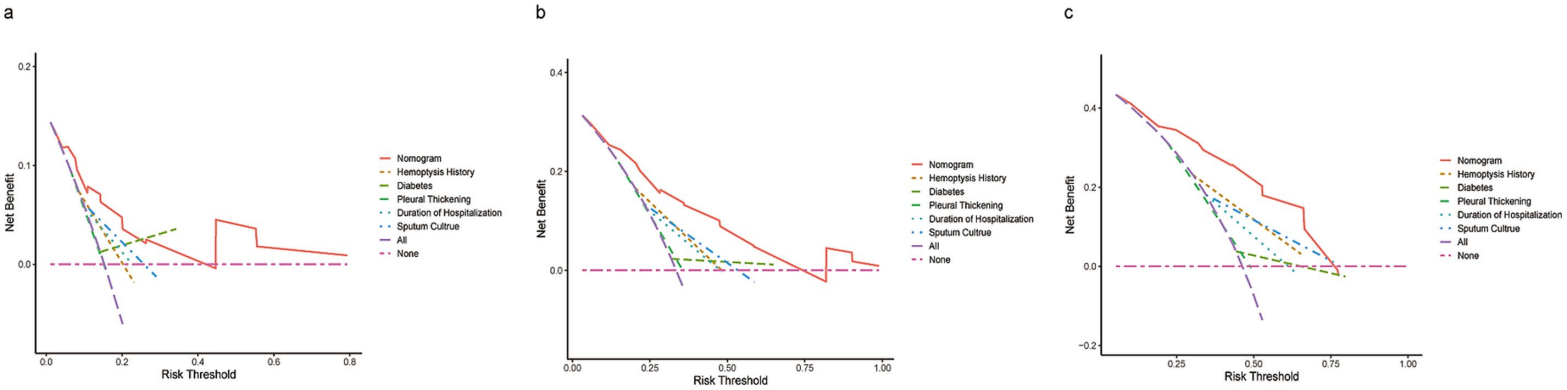
DCA of the nomogram compared to history of hemoptysis, diabetes, pleural thickening, duration of hospitalization, and sputum culture predicts 1-year **(a)**, 2-year **(b)**, and 3-year **(c)** recurrence. The purple dashed line indicates the net benefit of treating all patients, while the pink dashed line represents treating none. The y-axis shows net benefit, calculated as true positives minus false positives.

**Figure 6:**
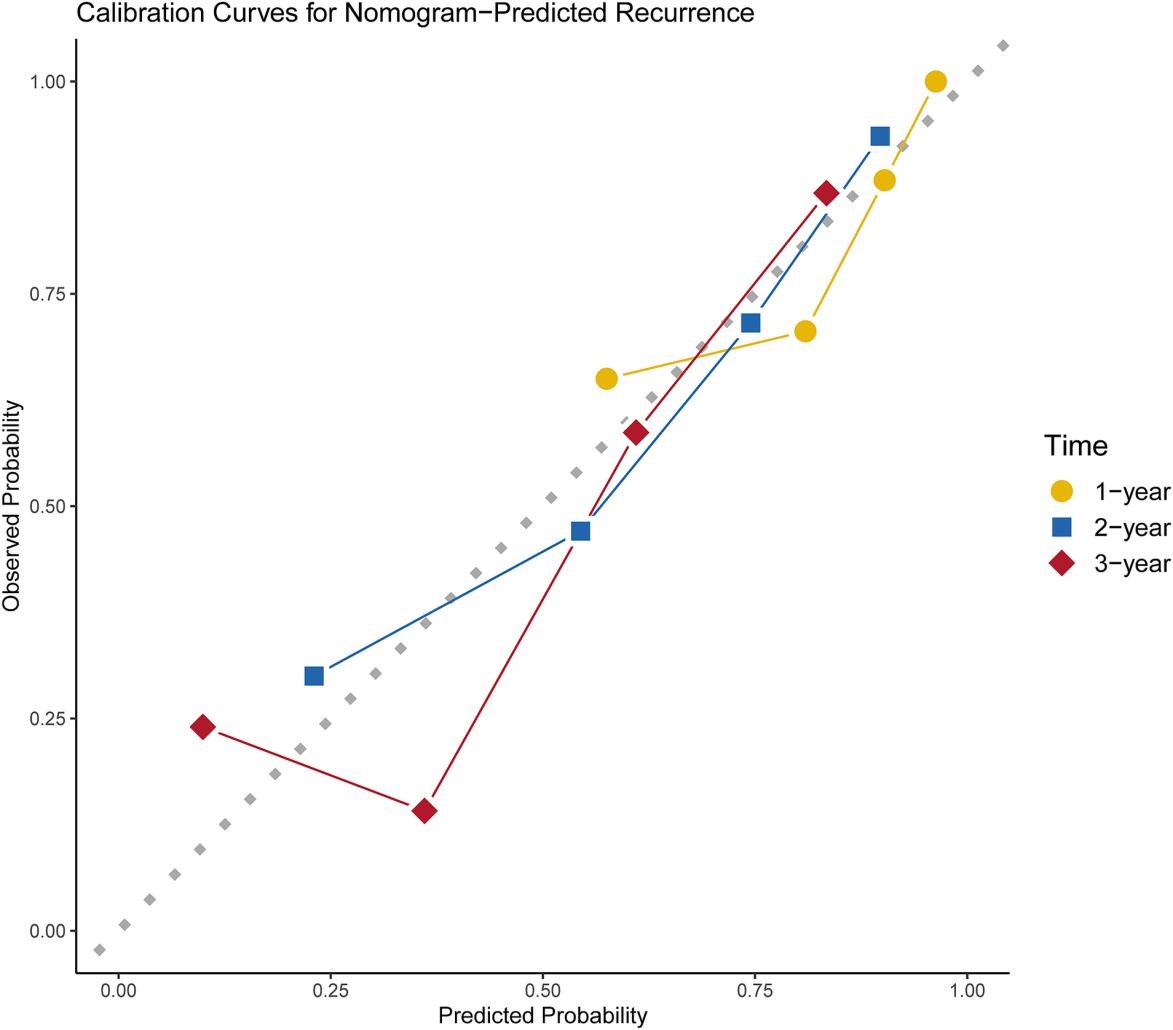
Calibration curves for 1-, 2-, and 3-year recurrence of nomogram predictions.

DCA and calibration plots were used to evaluate the nomogram’s net benefit and predictive accuracy. As shown in Figure 5, the DCA for 1-year, 2-year, and 3-year recurrence demonstrates that the nomogram consistently provides a higher overall net benefit compared to alternative models across most clinically relevant threshold probability ranges. Calibration analysis, as depicted in Figure 6, revealed a good agreement between predicted and observed risks, confirming the nomogram’s reliability in predicting recurrence at all three time points.

### Risk stratification based on the nomogram

**Figure 7:**
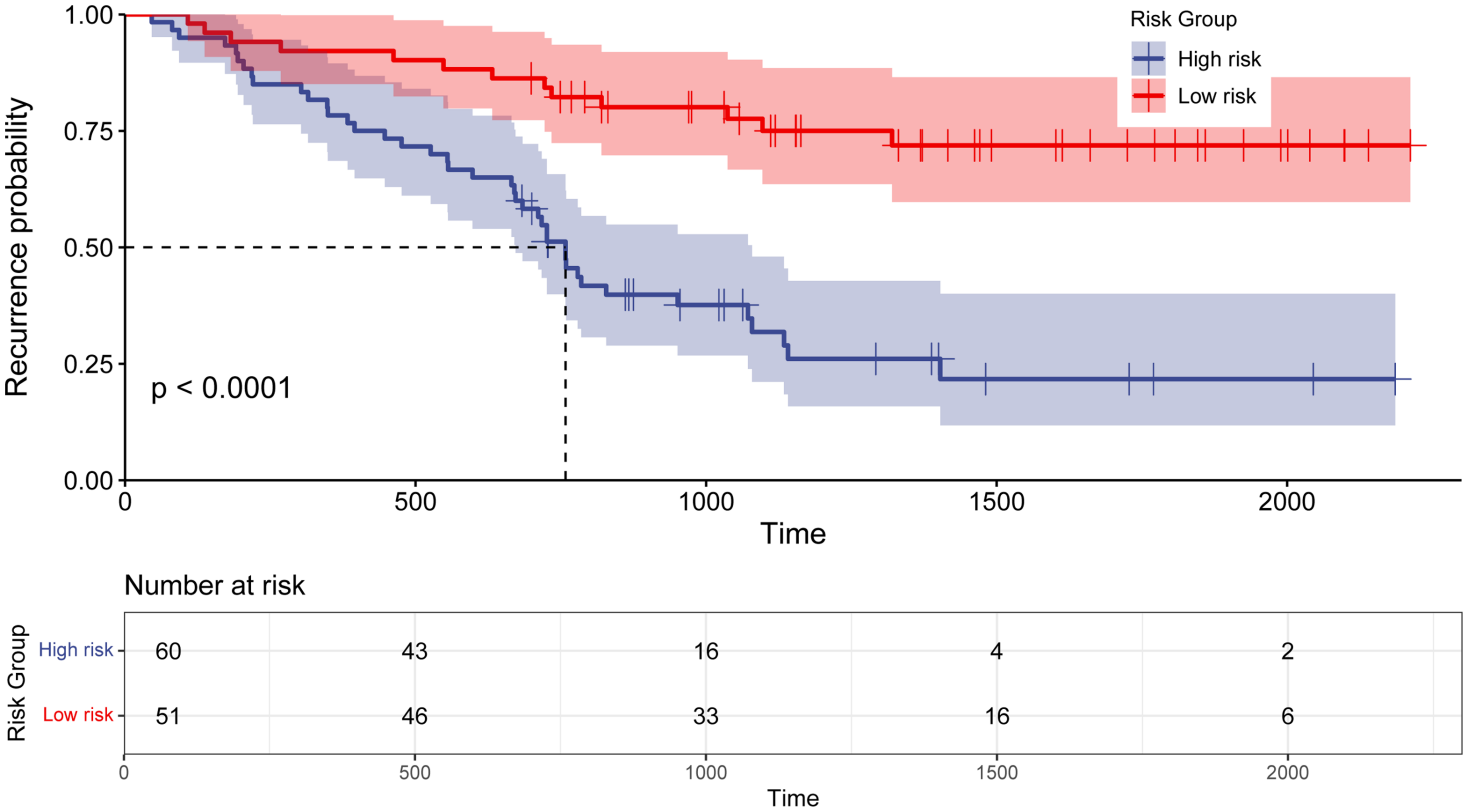
Kaplan–Meier curve for recurrence rate based on the prediction of nomogram. Low risk, Total points < 119 for recurrence probability; High risk, Total points≥ 119 for recurrence probability.

To evaluate the efficacy of the nomogram in stratifying CB patients into distinct risk categories, total points for each patient were calculated. The median score of 119 was used as the threshold to classify patients into low-risk and high-risk groups. Kaplan–Meier survival analysis was conducted to compare recurrence rates between the groups. A significant difference in median recurrence time was observed: the low-risk group had a median recurrence time of 1,239 days, while the high-risk group exhibited a significant shorter median recurrence time of 774 days (*p* < 0.0001; Figure 7).

## Discussion

Despite a high success rate (70–99%) for patients with acute or chronic recurrent hemoptysis undergoing initial BAE treatment, the recurrence rate of hemoptysis remains unchanged (3). Previous studies have identified several risk factors for hemoptysis recurrence after BAE in patients with bronchiectasis, and some evidence suggests that CB is associated with worse outcomes compared to non-cystic bronchiectasis. However, research on hemoptysis recurrence in CB patients after BAE remains scarce. This study aimed to investigate preoperative risk factors for hemoptysis recurrence in CB patients and to establish a predictive model that may assist in early risk stratification and individualized treatment planning.

In this retrospective cohort study, the preoperative characteristics of CB patients with hemoptysis treated by BAE were analyzed. History of hemoptysis, diabetes, pleural thickening, hospitalization duration ≥11 days, and positive sputum culture were identified as independent risk factors for hemoptysis recurrence. A predictive model was developed based on these five variables, all of which are routinely available in clinical practice. This model provides a practical tool for early risk stratification and individualized management of CB patients undergoing BAE.

Emerging evidence suggests that patients with diabetes frequently exhibit respiratory symptoms and are at increased risk for a variety of respiratory diseases (22, 23). In our study, diabetes was identified as a significant risk factor for recurrent hemoptysis in CB patients. The wide confidence interval observed for the HR of 15.0 (95% CI: 4.69–48.1) may be attributed to the limited sample size and low event rate in the diabetes-positive subgroup, which could have led to an unstable estimation of the HR. Nevertheless, this finding is consistent with previous studies indicating that diabetes impairs pulmonary arterial vasodilation, primarily due to exacerbated endothelial dysfunction (24). Additionally, diabetic microvascular and macrovascular complications had been shown to negatively impact pulmonary vasculature, underscoring a potential link between systemic vascular dysfunction and pulmonary pathology (22). Additionally, both microvascular and macrovascular complications of diabetes have been shown to adversely affect the pulmonary vasculature, highlighting a potential link between systemic vascular dysfunction and pulmonary pathology (2). Future studies with larger cohorts are warranted to validate these findings and enhance the precision of risk estimation.

*Pseudomonas aeruginosa* was the most frequently identified pathogen in sputum cultures (25), consistent with our findings, which showed its presence in 64.5% of sputum culture-positive patients. The predominance of *Pseudomonas aeruginosa* in sputum has been associated with an increased risk of acute exacerbations and increased hospitalization rates, as well as impaired health-related quality of life (HRQoL), diminished lung function, and heightened disease severity and mortality (26). Furthermore, the structural characteristics of CB facilitate mucus plug accumulation, increasing susceptibility to pulmonary infections and contributing to hemoptysis recurrence.

Prolonged hospital stays are associated with a higher risk of cross-infection, potentially worsening pulmonary disease progression and increasing the risk of hemoptysis recurrence, which is consistent with previous research (26). Moreover, prolonged hospitalization is often indicative of chronic pulmonary infections, during which certain inflammatory factors may contribute to bronchial artery regeneration, further heightening the likelihood of hemoptysis recurrence (27). A total of 99 patients (89.2%) exhibited pleural thickening greater than 3 mm in this study, significantly higher than in other studies (28). This finding suggested that CB patients may experience more complex pulmonary abnormalities and infection compared to non-CB patients. Furthermore, pleural thickening was often associated with an increased number of non-bronchial arteries, contributing to a high recurrence rate after bronchial artery embolization (BAE) (13, 29).

However, this study has several limitations. First, the retrospective design introduces inherent biases that cannot be fully eliminated. Second, the relatively small sample size, particularly the limited number of patients with certain risk factors, may have reduced the stability of the HR estimates. Larger, multicenter studies are needed to improve the accuracy and generalizability of the model. Finally, the model was internally validated; external validation using independent datasets is required to confirm its robustness and clinical applicability.

## Conclusion

This study developed a novel prognostic model based on clinical characteristics. Future large-scale, multicenter prospective studies are warranted to validate its clinical utility. The model may serve as an accessible and straightforward tool for prognostication, potentially improving clinical decision-making in patients with CB undergoing BAE.

## Data Availability

The raw data supporting the conclusions of this article will be made available by the authors, without undue reservation.
