# Supplementary material for: Development and validation of a nomogram for predicting hemoptysis recurrence in cystic bronchiectasis patients following bronchial artery embolization
Source: Front Med (Lausanne). 2025 May 15;12:1582008. doi: 10.3389/fmed.2025.1582008 (PMC12119253; doi:10.3389/fmed.2025.1582008)
Supplement: Supplementary file 1 [file Table_1.docx]

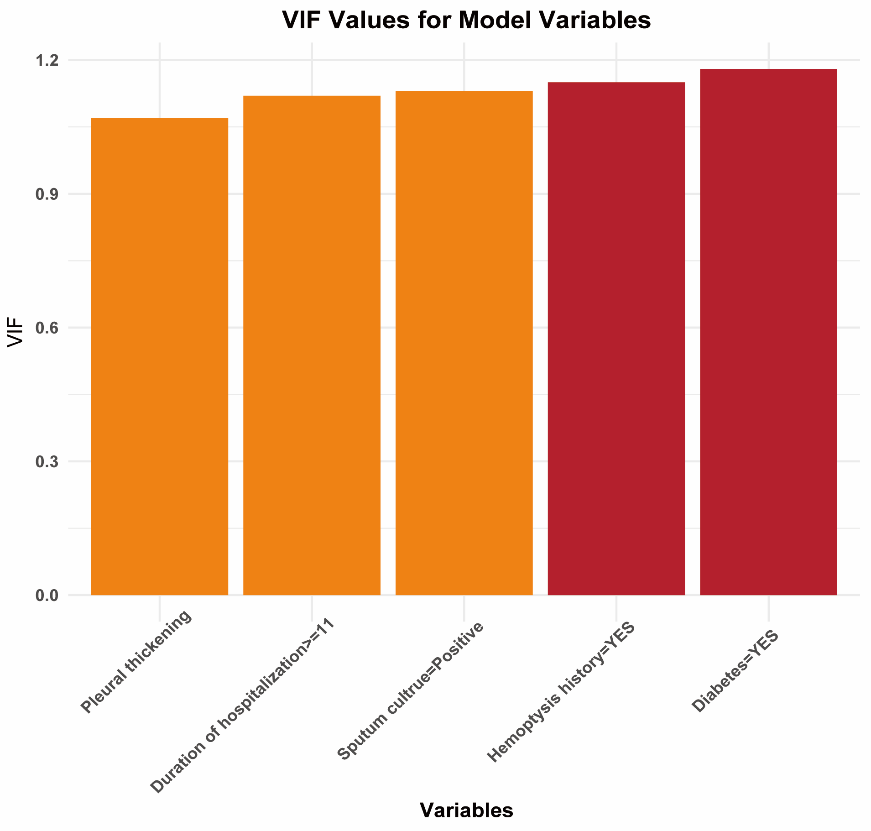


**Fig. S1** The VIF value of the variables enrolled in the nomogram model.


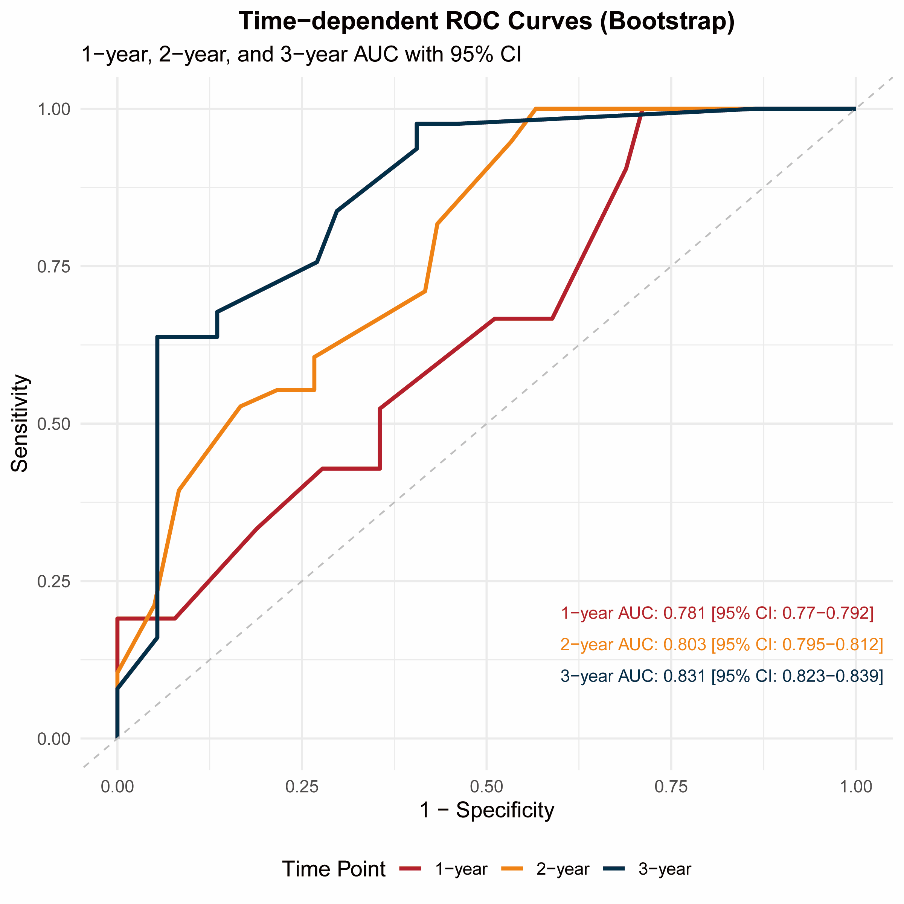


**Fig. S2** ROC curves of the nomogram for predicting 1-, 2-, and 3-year hemoptysis recurrence, based on 100 bootstrap resamples.

| Categorical variable | Cutoff value |
| --- | --- |
| Age(years) | 65 |
| SI | 200 |
| LOB: | 3 |
| CT bronchiectasis score | 10 |
| PLT(10^9^/L) | 162 |
| Duration of hospitalization(days) | 11 |
| NOC | 3 |
| LOC | 3.3 |
| CRP | 3.47 |
| WBC（10⁹/L） | 7.7 |
| NEUP | 0.77 |
| RBC (10^12^/L) | 4.0 |
| HGB (g/L) | 116 |
| Albumin(g/L) | 37.9 |
| TP(g/L) | 66.1 |
| PT(s) | 11.2 |
| APTT(s) | 26.9 |

***SI*** Smoking index; ***LOB*** Lobes of bronchiectasis; ***NOC*** Number of culprit bronchial arteries; ***LOC*** Length of culprit bronchial arteries; ***CRP*** C-Reactive protein; ***NEUP*** Neutrophil Percentage; ***RBC*** Red Blood Cell; ***HGB*** Hemoglobin; ***PLT*** Platelet; ***TP*** Total protein; ***PT*** Prothrombin time; ***APTT*** Activated partial thromboplastin time.

**Table S1** The cutoff values for continuous variables were determined based on receiver operating characteristic (ROC) curve analysis using the maximum Youden Index.
